# Shepherd’s Crook Curve: A Novel Technique for Angulated Side Branch Access in Bifurcation Angioplasty

**DOI:** 10.14797/mdcvj.1413

**Published:** 2024-08-06

**Authors:** Kanhai Lalani, M. Sudhakar Rao, Suheil Dhanse, Tom Devasia, Hashir Kareem, Ganesh Paramasivam

**Affiliations:** 1 Kasturba Medical College, Manipal; 2 Manipal Academy of Higher Education, Manipal, Karnataka, India; 3 Manipal Hospital, Bangalore, Karnataka, India; 4 Horizon Prime Hospital, Thane, Mumbai, Maharashtra, India; 5 KIMS Hospital, Trivandrum, Kerala, India

**Keywords:** shepherd’s crook curve, bifurcation angioplasty, reverse wiring

## Abstract

Coronary intervention involving the region of bifurcation remains a challenging issue for the cardiologist as well as a complication. A number of factors including the angulation of side branch with the main branch determines the success. Though provisional strategy remains the best option in bifurcation intervention, at times a two-stent strategy cannot be avoided. We report a case in which percutaneous coronary intervention was performed on the left anterior descending artery (LAD) at its bifurcation with a major diagonal branch (> 2.5 mm). The ostium of the diagonal was diseased, and the branch took off from the LAD at an unfavorable angle (> 120°). We describe the use of the “shepherd’s crook wire curve” approach, a modification of the reverse wire technique, which allowed us to successfully wire, dilate, and protect the diagonal and so named to reflect its resemblance to the shape of a shepherd’s crook.

## Background

Coronary intervention involving the region of a coronary artery bifurcation is complicated.^1^ The techniques used in bifurcation angioplasties are continuously evolving and often need to be tailored to the relevant anatomy of each specific patient. The selection of an optimal technique and the outcomes and complications of different techniques have been addressed in various trials. Currently, available evidence shows the absence of a clear benefit from the routine use of a two-stent strategy in bifurcation angioplasty.^2^,^3^,^4^,^5^,^6^,^7^ However, regardless of the strategy used, systematic wire placement in the side branch (SB) helps prevent SB occlusion. It also serves as a marker in the event of SB closure after main-branch stenting.^8^ Side branch access can be difficult in cases where the angle of take-off from the main branch is unfavorable for routine wiring techniques. In such a scenario, the use of a steerable tip catheter, for example the Venture Catheter (St. Jude Medical), or a dual lumen catheter has been reported.^9^,^10^,^11^ We report one such case in which the SB was accessed using a modification of the reverse wire technique reported by Kawasaki et al.^12^ We have dubbed this modification the “shepherd’s crook wire curve” technique to reflect the shape of the wire curve used.

## Case Presentation

A 63-year-old man presented with a 3-month history of exertional angina symptoms. An examination at presentation produced unremarkable findings. Testing of a serum sample for troponin generated negative results, and other routine biochemical and hematological parameters were within normal ranges.

**Figure 1:**
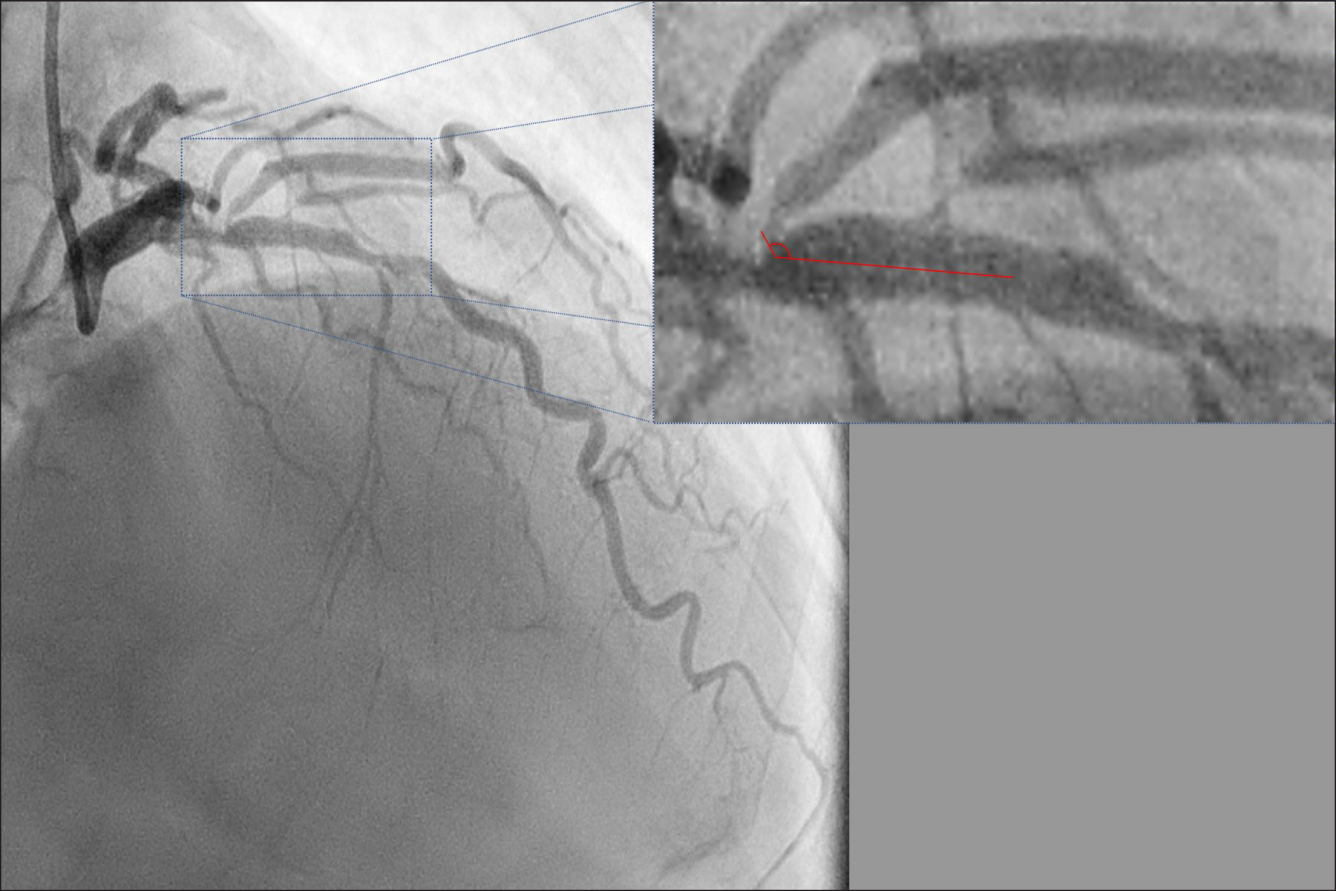
Diagnostic angiogram in right anterior oblique 40 cranial 40 view using a 6F catheter (radial access) showing a true bifurcation stenosis of the left anterior descending (LAD) diagonal junction. The ostium of the large diagonal is diseased and arises from the LAD at an angle of approximately 130° with the main branch (inset).

A coronary angiogram performed via transradial access revealed 80% stenosis of the proximal left anterior descending artery (LAD) across the ostium of the first (major) diagonal branch as well as an additional 50% stenosis across the major septal branch (Figure 1). The major diagonal also exhibited 90% ostio-proximal stenosis and originated from the main branch at an angle of approximately 130°.

**Figure 2:**
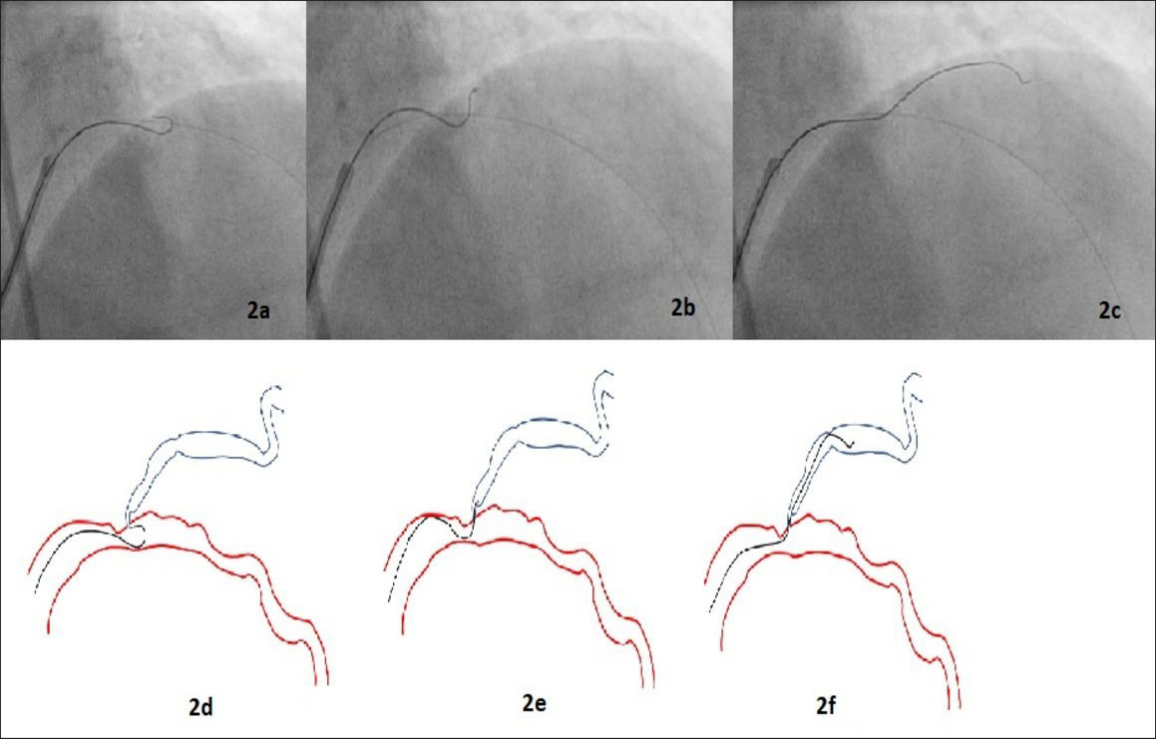
Angiogram images **(2a-c)** of percutaneous intervention **(2a)** through the femoral route showing advancement of the wire beyond the tip of the micro-catheter such that the wire assumed its pre-formed shape; **(2b)** the micro-catheter was withdrawn back into the guiding catheter, and with careful rotational manipulation the wire was pulled back until its tip engaged the ostium of the diagonal branch; **(2c)** once the wire tip entered the diagonal, the wire was advanced while torquing. Line diagram **(2d-f)** depicting the same information.

**Figure 3:**
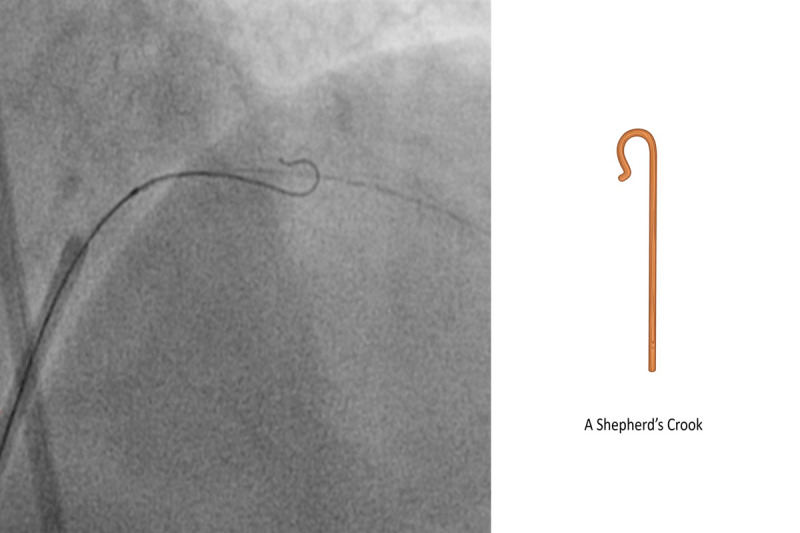
Diagnostic angiogram and line diagram image showing shepherd’s crook curve: beginning approximately 6 mm from the tip, a bend of 180° opposite the direction of the primary curve was introduced into the wire.

A 7F guiding catheter was used to cannulate the left main coronary artery trunk via transfemoral access. A Sion blue guidewire (Asahi Intecc) was passed into the distal LAD. To successfully wire the diagonal branch with an angulated take-off, we used a shepherd’s crook wire curve (Figure 2). A curve beginning approximately 6 mm from the tip was created in a polymer-jacket hydrophilic-coated guidewire—a Fielder XT-A wire (Asahi Intecc). A bend that was 180° opposite to the direction of the primary curve was introduced into the wire, 1 mm from the tip, so that the wire was shaped like a shepherd’s crook (Figure 3).

After the distal LAD was wired with a Sion wire, another Sion wire was passed into the LAD, and a microcatheter was positioned in the LAD distal to the lesion. The Sion wire in the microcatheter was exchanged for a preshaped Fielder XT-A wire, which was passed into the LAD. Distal to the lesion, the wire was advanced beyond the tip of the microcatheter so that the wire assumed its pre-formed shape. The microcatheter was withdrawn back into the guiding catheter. Then, with careful rotational manipulation, the wire was pulled back until its tip engaged the ostium of the diagonal branch.

**Figure 4:**
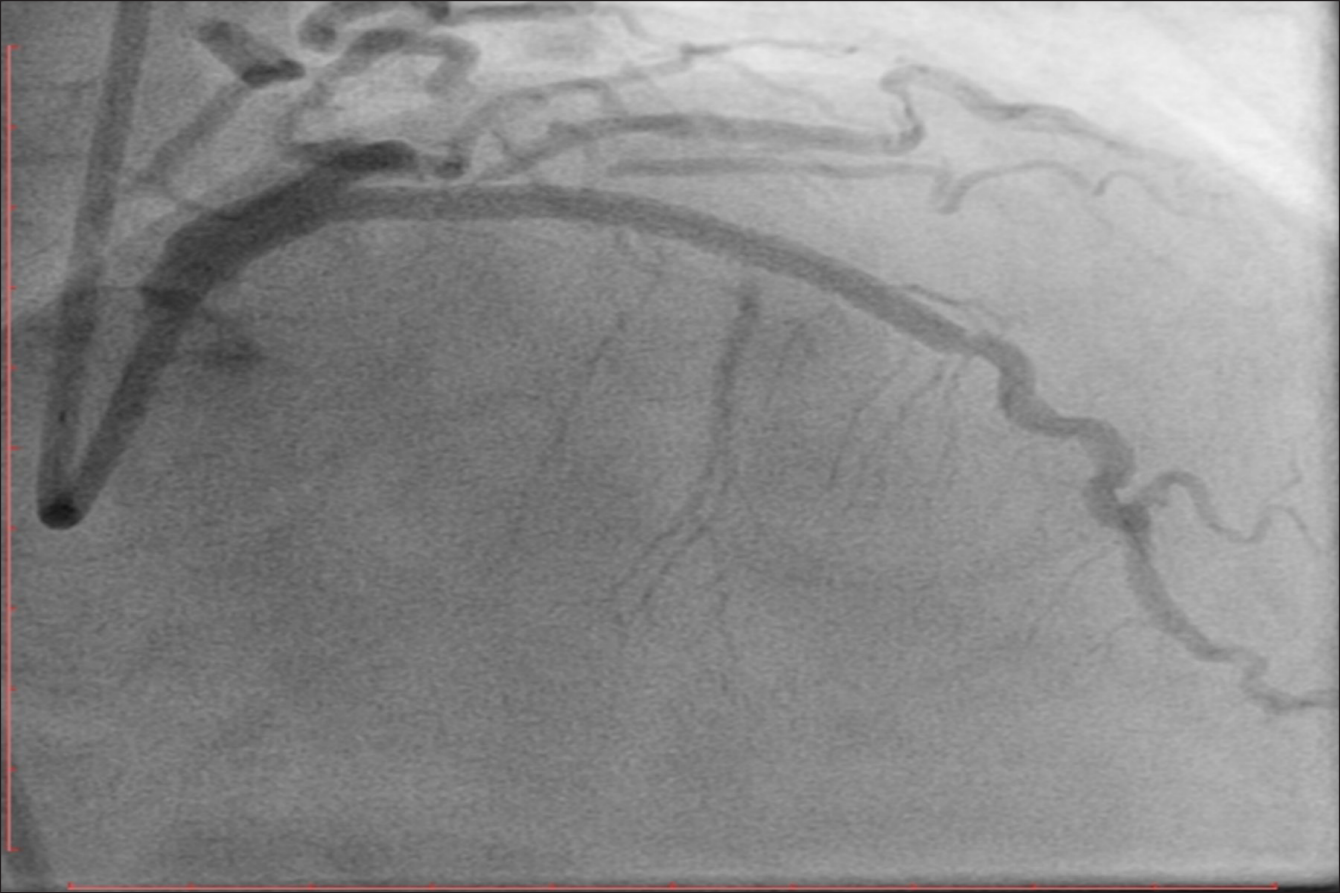
An angiogram in the right anterior oblique 40 cranial 40 view shows the final TIMI III flow in the left anterior descending artery after stenting and post dilatation with a non-compliant balloon.

Once the wire tip entered the diagonal, the wire was advanced while torquing. We then attempted to exchange this wire for a workhorse wire. The lesion at the diagonal ostium did not allow for the passage of the microcatheter into the diagonal. The microcatheter was removed using a 2-mm balloon to immobilize the Fielder XT-A wire, and the diagonal ostium was dilated using a 2-mm balloon. Subsequently, simultaneous kissing balloon dilatation of the diagonal and the LAD was performed. The Fielder XT-A was then exchanged for a Sion wire. The LAD was stented successfully with a 2.5-mm stent and post-dilated with a 3-mm noncompliant balloon (Figure 4). The procedure was completed without any complications. The final angiogram revealed an optimal result with TIMI III flow in the LAD and diagonal.

## Discussion

Angioplasty involving sites of bifurcation of a major coronary artery into two anatomically and physiologically significant branches are challenging.^1^ Acute loss or closure of a major branch during balloon dilation or stent deployment across the bifurcation may occur because of plaque or carina shift.^13^ The use of a provisional stenting strategy is the preferred strategy for most bifurcation lesions, and trials have shown worse outcomes when the SB has not been protected using a second wire.^1^,^14^ Often, the SB arises from the main branch at an angle unfavorable for wiring. Various techniques have been described to facilitate the passage of a guidewire into an angulated SB, including approaches involving a balloon back-stop, a deflectable tip catheter (for example, a Venture catheter) or microcatheter-facilitated reverse wiring.^9^,^10^

Our patient’s coronary angiogram showed true bifurcation stenosis of the proximal LAD across the ostium of a major diagonal branch with an angulated take-off (at an angle of approximately 130°). In addition, the ostium of the SB lay on the distal shoulder of the proximal LAD lesion, resulting in a significant wire bias. Multiple attempts to access the diagonal branch using routine techniques failed. The diagonal was successfully wired using our modification (shepherd’s crook curve) of the reverse wire technique first described by Kawasaki et al.^12^

Several techniques to aid the passage of guidewires into angulated SBs have been described in the literature. The reverse wire technique involves creating a hairpin bend 5 cm from the tip of a wire.^12^ Watanabe et al. reported that for an in vitro bench test of the reverse wire technique, the best results were obtained when a polymer-coated hydrophilic wire was used.^11^ In this approach, the wire, which is bent and pre-shaped outside, is inserted via the guiding catheter into the coronary artery distal to the ostium of the angulated SB. Subsequently, the wire is manually steered back into the SB. Once the SB is dilated via serially increasing balloon dilatations, the wire is exchanged for a straight wire and the angioplasty is completed. The application of this technique requires a large main branch to allow for maneuvering of the wire. Moreover, these authors believe that there is a risk of wire entrapment and SB ostial dissection during wire manipulation when this technique is used.^11^

A twin-pass catheter (eg, a Crusade catheter) has also been used in this context. A wire positioned in the main branch is used to position the exit port of the dual-lumen catheter at the ostium of the SB. This approach provides additional support while wiring the SB and can somewhat reduce wire bias.^15^ A catheter with a deflectable tip (eg, a Venture catheter) may also be used to angle the wire into the SB.^9^,^10^ The “balloon back-stop” technique involves the inflation of a balloon at low pressure immediately distal to the take-off of the SB and use of this balloon to deflect a curved wire into the ostium of the SB.^16^ Although this approach has not been systematically studied in trials, there exists a theoretical risk of producing a dissection at the site of balloon inflation, and this technique entails the use of additional hardware. A deflecting tip guidewire such as the Steer-IT Deflecting Tip guidewire (Cordis Corporation) also may be used. The tip of the wire can be deflected using the mechanism at the proximal end of the wire.^16^

In our patient, the ostium of the SB lay on the distal shoulder of the proximal LAD lesion, resulting in a significant wire bias. This in combination with the extremely angulated ostium of the SB precluded the possibility of success with conventional techniques. Also, the lack of availability of a deflectable tip guidewire or the Venture catheter contributed to the need for improvisation. Therefore, we decided to attempt the reverse wire technique. However, the diagonal branch was a tortuous vessel, and we felt that advancing the bent wire across the tortuosity carried a significant risk of dissection. Therefore, we decided to modify the technique by using a shorter and smoother curve instead of a hairpin bend. We used various wires, including hybrid wires such as Sion Blue (0.5 gm tip load), as well as hydrophilic wires such as Whisper (non-tapered, polymer jacketed, 1.0 gm tip load) and Fielder FC (non-tapered, polymer jacketed, 0.8 gm tip load). However, none of these wires with their respective curves were able to navigate the angulated diseased opening of the SB.

After trying different wires, we eventually used the Fielder XT-A (tapered, polymer jacketed, hydrophilic, 1 gm tip load) and successfully maneuvered the wire distally with a shepherd’s crook curve. We believe that this technique can be carried out using any hydrophilic polymer jacketed wire with 1:1 torquability, flexibility, trackability, and shape retention properties.

## Conclusion

The use of a shepherd’s crook curve is a simple, reproducible, and, most importantly, customizable technique for accessing an angulated SB. The wire may be shaped in accordance with the relevant anatomy, and the curve may be modified to suit the angle in question. This technique also reduces procedure costs because it does not require the use of additional hardware.
